# Building capacity for implementation—the KT Challenge

**DOI:** 10.1186/s43058-021-00186-x

**Published:** 2021-07-28

**Authors:** Agnes T. Black, Marla Steinberg, Amanda E. Chisholm, Kristi Coldwell, Alison M. Hoens, Jiak Chin Koh, Allana LeBlanc, Martha Mackay, Amy Salmon, M. Elizabeth Snow

**Affiliations:** 1 Providence Health Care, 1190 Hornby St, Suite 409G, Vancouver, BC V6Z 2K5 Canada; 2 Evaluation & KT Consultant and Educator, 3037 West 13th Ave, Vancouver, BC V6K 2V1 Canada; 3 Vancouver Coastal Health Research Institute, 6/F, 2635 Laurel St, Vancouver, BC V5Z 1M9 Canada; 4 Transplant Research Foundation of BC, 555 W 12th Ave 3rd floor, Vancouver, BC V5Z 3X7 Canada; 5 Providence Health Care and University of British Columbia, 1081 Burrard St, Vancouver, BC V6Z 1Y6 Canada; 6 Providence Health Care, 1081 Burrard St, Vancouver, BC V6Z 1Y6 Canada; 7 Vancouver Coastal Health, 899 W 12th Ave, Vancouver, BC V5Z 1M9 Canada; 8 Centre for Health Evaluation & Outcome Sciences, 588 – 1081 Burrard Street, St. Paul’s Hospital, Vancouver, BC V6Z 1Y6 Canada

**Keywords:** Capacity building, Evidence-based practice, Knowledge translation, Health care professionals, Implementation science

## Abstract

**Background:**

The KT Challenge program supports health care professionals to effectively implement evidence-based practices. Unlike other knowledge translation (KT) programs, this program is grounded in capacity building, focuses on health care professionals (HCPs), and uses a multi-component intervention. This study presents the evaluation of the KT Challenge program to assess the impact on uptake, KT capacity, and practice change.

**Methods:**

The evaluation used a mixed-methods retrospective pre-post design involving surveys and review of documents such as teams’ final reports. Online surveys collecting both quantitative and qualitative data were deployed at four time points (after both workshops, 6 months into implementation, and at the end of the 2-year funded projects) to measure KT capacity (knowledge, skills, and confidence) and impact on practice change. Qualitative data was analyzed using a general inductive approach and quantitative data was analyzed using non-parametric statistics.

**Results:**

Participants reported statistically significant increases in knowledge and confidence across both workshops, at the 6-month mark of their projects, and at the end of their projects. In addition, at the 6-month check-in, practitioners reported statistically significant improvements in their ability to implement practice changes. In the first cohort of the program, of the teams who were able to complete their projects, half were able to show demonstrable practice changes.

**Conclusions:**

The KT Challenge was successful in improving the capacity of HCPs to implement evidence-based practice changes and has begun to show demonstrable improvements in a number of practice areas. The program is relevant to a variety of HCPs working in diverse practice settings and is relatively inexpensive to implement. Like all practice improvement programs in health care settings, a number of challenges emerged stemming from the high turnover of staff and the limited capacity of some practitioners to take on anything beyond direct patient care. Efforts to address these challenges have been added to subsequent cohorts of the program and ongoing evaluation will examine if they are successful. The KT Challenge program has continued to garner great interest among practitioners, even in the midst of dealing with the COVID-19 pandemic, and shows promise for organizations looking for better ways to mobilize knowledge to improve patient care and empower staff. This study contributes to the implementation science literature by providing a description and evaluation of a new model for embedding KT practice skills in health care settings.

Contributions to the literature

- Disseminates information about a promising and innovative KT capacity-building program/model
- Characterizes unique elements of the program including its focus on health care professionals who have responsibility for implementing practice changes, a multi-component support model, low cost, and applicability to a variety of professionals in diverse practice settings
- Provides evidence from early evaluation of improvements in knowledge, confidence, and ability to implement practice change

## Background

There is an urgent need to reduce the gap between the creation of evidence and its implementation into practice. However, alarmingly, up to 70% of all organizational improvement efforts fail, including those in health care settings [1]. To address this shortcoming, knowledge translation (KT) initiatives and training programs in health care have proliferated in recent years [2–7]. Key barriers to successful KT include lack of knowledge, skills, time, and leaders’ endorsement, and competing priorities of organizations [8–10]. Important enablers include health care professionals’ (HCPs) positive beliefs about the benefits of participation in training programs, combined with expert guidance and organizational support [11]. HCPs want to provide the best care possible, but many lack the knowledge and skills to effectively move evidence into practice. A 2012 survey of health care providers, administrators, and researchers revealed that nearly 80% of respondents wanted to improve their knowledge and skills related to implementation [10]. Moreover, research has demonstrated that high-quality implementation strategies are associated with successful implementation [11], thus supporting the need to offer KT and implementation capacity building, training, and support to clinicians. A recent systematic review stressed the importance of increasing the number and reach of training opportunities to address the lack of dissemination and implementation training options [12].

Providing KT training takes a dedicated effort from health care organizations. Much of the research conducted on how to close the evidence-to-practice gap has been led by and/or focused on academic researchers with minimal involvement from those who provide or use health services [3]. First-generation interventions used co-creation models. For example, a program in the UK used a partnership approach to KT and reported that implementation projects co-produced by researchers and end users were more likely to successfully adapt new evidence into practice, and the co-production experience encouraged future collaboration between the parties [3]. Researchers and health care leaders have called for training that builds capacity among those working at the point of care [4–6], who often have the responsibility to implement, but do not have adequate skills and support to do so effectively. Involving those who use research knowledge in efforts to implement it has multiple benefits, including enhancing the effectiveness of implementation efforts [3]. Eames and colleagues, for example, found changes in clinician-reported behavior, especially the use of strategies for implementing a change in practice, following participation in their KT capacity-building intervention for occupational therapists, as well as changes in the culture to one in which clinicians engaged in KT as part of their clinical practice [2]. Similarly, a longitudinal evaluation of a program for implementers, called Practicing KT, showed increased use of, knowledge of, and self-efficacy in KT among those who completed the program [9].

Many programs exist to increase KT capacity, including some that offer training in implementation skills, but there is lack of information on their effectiveness. Programs that use active forms of learning to promote the acquisition of KT skills have been shown to be effective in transferring knowledge, but there is scant evidence of sustained practice change resulting from these sessions [7–9, 13]. KT scholars have acknowledged the need for longitudinal evaluation of KT capacity-building initiatives to assess the sustainability of outcomes from these programs [4, 9].

Armed with these findings, we developed an implementation support program for HCPs, aimed at building capacity to move research evidence into practice. The program, called the “KT Challenge,” was modeled on a similar research capacity-building initiative for clinicians. That program, in place at our organizations for more than 10 years, has been shown to be effective in supporting practice changes, enhancing evidence-based practice, and increasing interest in research engagement, including increased interest in graduate school [14, 15]. The KT Challenge program was implemented at two health organizations in British Columbia, Canada, and offered to all HCPs employed at the organizations. We report an evaluation of the KT Challenge program with respect to uptake by HCPs, impact on KT capacity (knowledge, skills, and confidence) of HCPs, and impact on evidence-based practice changes.

## Methods

### Overview of the KT Challenge program

The KT Challenge is a multi-component implementation support program that involves training, funding, peer review, and mentorship. The key components of the KT Challenge program are briefly described below.

#### Letter of Intent (LOI)

In the LOI, teams identify the practice change they want to implement, document the need for this change in their practice context, and summarize evidence of its effectiveness. LOIs also require the signature of a manager to ensure management support and endorsement, and the identification of team members. The LOIs are formatively reviewed and, in keeping with the capacity-building approach of this program, revisions are suggested when required. Some teams are screened out of the program at this point if their proposed LOI does not identify an evidence-based practice change, but most teams are given feedback and invited into the next stage.

#### Workshops

Teams attend two half-day workshops, focusing on developing an implementation plan, and evaluating the effectiveness of the implementation of the practice change. Topics include implementation theories and frameworks, stakeholder engagement, identifying barriers and facilitators, implementation strategies, and evaluation planning.

#### Mentorship

Teams are supported to find a mentor within their clinical area to support the implementation of the practice change and assist with navigating facilitators and barriers. Mentors are also invited to participate in the evaluation of the KT Challenge program throughout the project.

#### Online resources

Teams have access to an online learning site where a curated set of readings and resources is posted.

#### Management support

Teams are required to obtain their manager’s support for conducting the implementation project and dedicating time to it.

#### Funding

Successful teams are awarded $5000 to cover costs related to personnel, materials and supplies, stakeholder engagement, and services.

#### Program leads

The KT Challenge is run by a program lead from each organization. The leads promote the program, coordinate the review processes, monitor team progress, support teams to successfully navigate barriers, and lead the evaluation of the program.

**Fig. 1:**
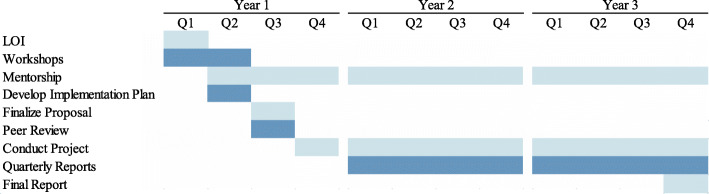
Timeline for the KT Challenge program. Timeline for the program, years 1 through 3

Figure 1 outlines the timeline of activities for each cohort. Funded teams are asked to submit a quarterly report to provide an update on their progress, as well as a final report on the impact of their project on the intended practice changes.

### Evaluation plan

To evaluate the KT Challenge, we used a mixed-methods retrospective pre-post design [15, 16] to assess the impact of the program on workshop participants and funded team members. The evaluation focused on the program uptake and changes in KT capacity (knowledge, skills, and confidence) among HCPs, and the impact on practice change. Online surveys (including both Likert scale and open-ended response questions) were used to collect data at four time points: after each of the two workshops, 6 months into implementation, and at the end of their 2-year funding. Only team leaders and mentors of funded teams complete the 6-month and end-of-project surveys. The online surveys collect both quantitative and qualitative data on participants’ knowledge, confidence, and ability. The 6-month check-in survey assessed knowledge, confidence, and ability by asking participants to rate their (1) overall knowledge of how to support practice changes, (2) level of confidence in supporting practice changes, and (3) ability to support practice changes on a 5-point scale that ranged from low (1) to high (5) before the program and 6 months into their projects. In this paper, we only consider data from the 6-month check-in survey because it captures a robust operationalization of KT capacity (knowledge, confidence, and ability) during the actual implementation of the practice changes and offers a larger sample size than the end-of-project survey which had only been completed by one cohort. Data on the impact of the practice change is gleaned from the final reports submitted by the team leads.

### Data analysis

The qualitative data were analyzed using a general inductive approach, which involved the identification of themes and sub-themes related to the quality of the program, usefulness of various program components, impact on participants, impact on practice changes, and challenges. The themes were initially identified by one of the authors and then reviewed by two other authors. The quantitative data were analyzed in Excel (Microsoft Corporation), using the Wilcoxon signed-ranks test to compare pre- and post-scores on self-ratings of knowledge, skills, confidence, and ability. Statistical significance was set at *p* < 0.05.

## Results

### Uptake of the KT Challenge by practitioners

**Fig. 2:**
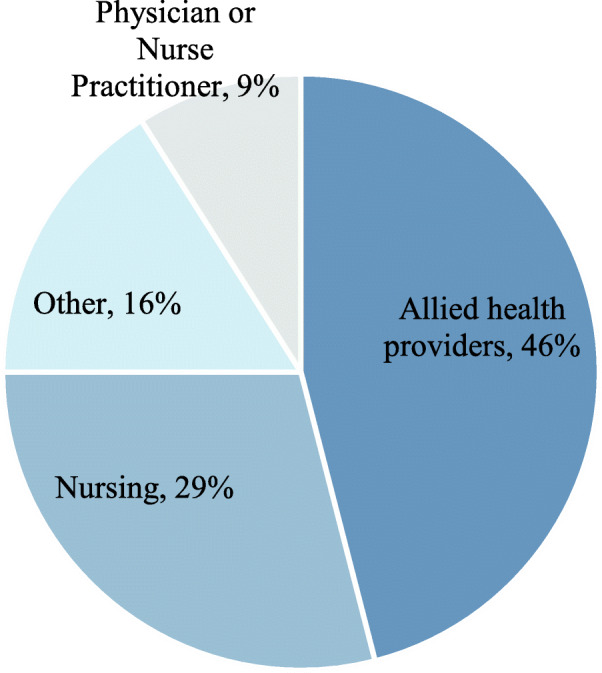
KT Challenge program participants by profession. Program participants included nurses, physicians, nurse-practitioners, allied health, and others. The “other” category included peer outreach workers, Aboriginal patient navigators, youth care workers, research scientists, overdose prevention specialists, etc.

Four cohorts of participants have taken part in the program, with a fifth cohort recently launched. The first cohort (2016) has completed their projects. To date, 24 teams have been funded, comprising 185 HCPs. Participants have included a wide range of HCPs involving 23 types of practitioners working within a range of practice settings (see Fig. 2).

Interest in the program has remained steady, with 13 teams participating in cohort 3 (6 were funded). While 9 teams initially joined cohort 4, the number of teams dropped significantly when the COVID-19 pandemic struck, and only 2 teams submitted funding proposals. However, cohort 5 was launched in October 2020, and 13 teams applied to participate, including teams proposing COVID-related projects.

### KT capacity

The average survey response rate from the three cohorts was 76% for post-workshop #1 survey, 59% for post-workshop #2 survey, and 100% for both the 6-month check-in survey and the end-of-project survey.

**Fig. 3:**
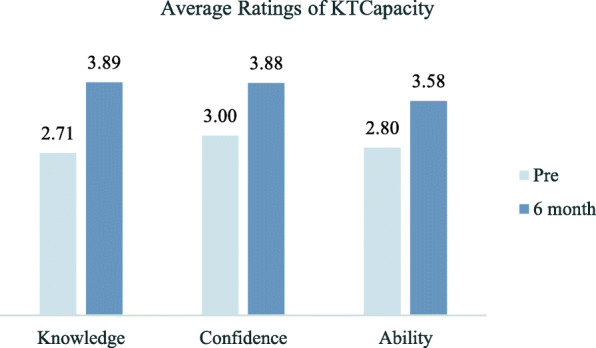
Average ratings of KT capacity at baseline compared to the 6-month follow-up. Rated on a 5-point scale with 1 representing “low” and 5 representing “high”

Participants reported statistically significant increases in knowledge and confidence at the 6-month mark of their projects, compared with before the initiative (*p* < 0.05). In addition, at the 6-month check-in, practitioners reported statistically significant increases in their ability to implement practice changes (Fig. 3).

Numerous comments were provided on the surveys and in the final reports attesting to the knowledge, skills, and confidence acquired to effectively support implementation:

*Thinking about addressing barriers and not simply providing MORE information and MORE education has stuck with me and has impacted how I approach other initiatives and projects.*

*Changing how I think about implementing or addressing any practice change. I am constantly thinking about what REAL barriers to change might be.*

### Impact on practice change

Data on the impact of the program on practice changes is available from the first cohort only, as funded projects in subsequent cohorts are still in progress. Eight teams were funded in the first cohort, but two were unable to complete their projects due to management changes and shifting priorities within their departments. Of the remaining six teams, three showed demonstrable practice changes across their respective practice areas, giving the program a 50% success rate:

1. In a cardiac inpatient unit, depression screening increased from none to 75%, and follow-up discussions with primary care providers were recorded at 36%.
2. In a tertiary hospital setting, malnutrition screening increased by 50%.
3. In a hospital-based physical rehabilitation program, 67% of physical therapists and occupational therapists reported increased uptake of the Canadian Stroke Best Practice Recommendations.

Three teams completed their projects but were not able to demonstrate measurable practice change. Cohort 2 (*n* = 9 teams) and cohort 3 (*n* = 6 teams) are currently completing their projects, with promising results, including one team that has significantly increased the percentage of patients with spinal cord injury receiving functional electrical stimulation treatment. Another team has garnered strong interest in their topic—treating problematic methamphetamine use—and was approached by local health care policymakers as well as staff from the United States Office of National Drug Control Policy, wanting information on their implementation plan.

### Reported challenges

Despite the positive impacts on practitioners’ KT capacity and demonstrable practice changes, many teams experienced challenges, common to most KT programs [5, 6, 13], and including:

- Team member turnover
- Manager turnover
- Gaps in communication with mentor
- Projects taking more time than anticipated
- Lack of support from key stakeholders for the practice change

Steps were taken in response to these challenges, as outlined below.

## Discussion

The evaluation of the KT Challenge program demonstrates that a multi-faceted implementation support program for HCPs is effective at moving evidence into practice and can be conducted within a modest budget ($5000 per team). The content of the KT Challenge workshops is designed to address the identified barriers to successful implementation of evidence into practice, including lack of knowledge, skills, time, and leaders’ endorsement [8–10]. The steady numbers of applicants for the KT Challenge program indicate sustained interest, including in the most recent cohort (October 2020), in the midst of the global pandemic, 13 teams submitting LOIs to join the program. This confirms the findings of the 2012 survey that showed 80% of health care respondents are interested in improving their knowledge and skills related to implementation [10].

Among challenges faced by teams, lack of time, changes in personnel, and shifting work priorities were the most commonly cited. The following steps were taken to improve the program for future cohorts:

- Program leads provide feedback and suggestions to teams in response to their quarterly reports
- Workshops and program documents were revised to specifically highlight the need for the project evaluation to collect data on the uptake of the practice changes
- Program leads undertook more promotion of the program within their organizations to strengthen management’s commitment to the funded teams

However, even the teams who were unable to complete, or whose projects did not lead to measurable practice change, agreed that participation in the program was beneficial. We had hoped to see all projects result in demonstrable practice change arising from their interventions, but given the many barriers to practice improvements, it is unreasonable to expect 100% success. As Durlak and Dupre have noted, “Expecting perfect or near-perfect implementation is unrealistic. Positive results have often been obtained with levels around 60%...no study has documented 100% implementation for all providers.” (19, p. 331). In comparison, 50% of our completed teams reported evidence-based practice changes, demonstrating the effectiveness of the KT Challenge program. Furthermore, the 185 HCPs who participated in the program gained knowledge and confidence in KT practice skills, as well as improved ability to implement practice change.

We revised the KT Challenge program to enhance the relevance of the projects to patients and families and to improve the dissemination of the findings to audiences that include patients and families. Beginning with the 2019–2020 cohort, all KT Challenge teams were required to include a Patient-Family Partner on their implementation team, and all funding proposals are now reviewed by a panel that includes Patient-Family Partners.

Our evaluation shows that the KT Challenge has been very effective at increasing capacity in KT skills and has provided information on areas where the program can be improved. We have carefully reviewed evaluation comments from each cohort in the KT Challenge and have made adjustments to the program based on feedback from participants, offering progressively improved support with each cohort. We hope this support will lead to a higher percentage of teams demonstrating measurable practice changes.

These findings are based on the first cohort of teams who completed the entire program, which limited our ability to do more fulsome comparisons between the teams who were able to demonstrate practice improvements and those who were not able to complete their projects.

## Conclusions

The KT Challenge program is a promising initiative that can be adopted across a variety of clinical settings to support the effective uptake of evidence-based practice. This evaluation demonstrates that clinicians will respond to opportunities for KT training, that enhanced capacity for KT skills is achievable with support, and—most importantly—that successful practice change can result from small-scale, mentored, and funded KT projects in clinical practice settings. This evaluation study contributes to the implementation science literature by providing a description of a new and effective model for embedding KT practice skills in health care settings.

## Data Availability

The datasets used and/or analyzed during the current study are available from the corresponding author on reasonable request.

## Abbreviations

HCP: Health care professional
KT: Knowledge translation
LOI: Letter of Intent
OT: Occupational therapist
PT: Physiotherapist
